# Identification of Cyclic Dipeptides from *Escherichia coli* as New Antimicrobial Agents against *Ralstonia Solanacearum*

**DOI:** 10.3390/molecules23010214

**Published:** 2018-01-19

**Authors:** Shihao Song, Shuna Fu, Xiuyun Sun, Peng Li, Ji’en Wu, Tingyan Dong, Fei He, Yinyue Deng

**Affiliations:** 1 State Key Laboratory for Conservation and Utilization of Subtropical Agro-Bioresources, South China Agricultural University, Guangzhou 510642, China; lklssh@stu.scau.edu.cn (S.S.); fu_shuna@163.com (S.F.); sunxiuyun1998@163.com (X.S.); tydong95@outlook.com (T.D.); 2 Guangdong Innovative Research Team of Sociomicrobiology, College of Agriculture, South China Agricultural University, Guangzhou 510642, China; 3 Integrative Microbiology Research Centre, South China Agricultural University, Guangzhou 510642, China; hefei@scsio.ac.cn; 4 School of Biological and Science Technology, University of Jinan, Jinan 250022, China; dayuleepong@163.com; 5 Department of Chemistry, National University of Singapore, Science Drive 3, Singapore 117543, Singapore; chmwujie@nus.edu.sg

**Keywords:** bacterial wilt, *Ralstonia solanacearum*, biocontrol agent, antimicrobial activity, cyclic dipeptides

## Abstract

*Ralstonia solanacearum* is a causative agent of bacterial wilt in many important crops throughout the world. How to control bacterial wilt caused by *R. solanacearum* is a major problem in agriculture. In this study, we aim to isolate the biocontrol agents that have high efficacy in the control of bacterial wilt. Three new bacterial strains with high antimicrobial activity against *R. solanacearum* GMI1000 were isolated and identified. Our results demonstrated that these bacteria could remarkably inhibit the disease index of host plant infected by *R. solanacearum*. It was indicated that strain GZ-34 (CCTCC No. M 2016353) showed an excellent protective effect to tomato under greenhouse conditions. Strain GZ-34 was characterized as *Escherichia coli* based on morphology, biochemistry, and 16S rRNA analysis. We identified that the main antimicrobial compounds produced by *E. coli* GZ-34 were cyclo(l-Pro-d-Ile) and cyclo(l-Pro-l-Phe) using electrospray ionization mass spectrometry (ESI-MS) and nuclear magnetic resonance (NMR) analysis. The two active compounds also interfered with the expression levels of some pathogenicity-contributors of *R. solanacearum*. Furthermore, cyclo(l-Pro-l-Phe) effectively inhibited spore formation of *Magnaporthe grisea*, which is a vital pathogenesis process of the fungal pathogen, suggesting cyclic dipeptides from *E. coli* are promising potential antimicrobial agents with broad-spectrum activity to kill pathogens or interfere with their pathogenesis.

## 1. Introduction

Bacterial wilt is a systemic, infective, and destructive soil-borne disease caused by *Ralstonia solanacearum*, commonly known as “plant blast”. It is an important plant disease in tropical, subtropical, and warm regions [1]. *R. solanacearum* has an unusually wide host range, infecting more than 200 species belonging to more than 54 botanical families, including economically-important crops [2,3,4]. It represents a heterogeneous group subdivided into five races based on host range, five biovars based on physiological and biochemical characteristics [5,6], and four phylotypes roughly corresponding to geographic origin [7,8]. It can survive in soil for many years and can spread through water, rhizosphere contact, and farming [9]. It usually parasitizes the vascular tissue of plants, and mainly invades from the root or stem of the host plant [10]. The fast proliferation and propagation of pathogen cells causes plant duct expansion and ultimately leads to whole plant wilting and death [11]. 

There are still no effective strategies to control the plant diseases caused by *R. solanacearum*. Bacterial wilt continues to be one of the most serious problems for field-grown crops in many regions around the world [12,13]. At present, the major prevention and cure method for bacterial wilt is chemical control, but chemical pesticides cannot effectively prevent the occurrence of the disease. The other sequela of long-term usage of chemical pesticides are the emergence of drug-resistant bacteria and serious environmental pollution [14]. Alternatively, screening of bacterial wilt-resistant cultivars is considered a promising approach [15]. However, it is difficult to develop resistant cultivars against *R. solanacearum* in many crops, reducing the effectiveness of this strategy.

Biological control is one effective method to control plant disease due to its advantages of limiting the production of drug-resistant bacterial pathogen [16]. It is the use of biocontrol agents themselves or their active substances in the prevention and control of plant diseases [17]. It is a way to adapt to the trend of “green agriculture”. Many biocontrol agents have been isolated and tested for their activity against bacterial wilt, including *Rhizophagus irregularis* [18], *Pseudomonas fluorescens* [19], *Bacillus amyloliquefaciens* [20], and *Stenotrophomonas maltophilia* [21]. In this study, we isolated and identified three new bacterial species with high activity against *R. solanacearum*. Two antimicrobial compounds, cyclo(l-Pro-d-Ile) and cyclo(l-Pro-l-Phe), were identified to be produced by the biocontrol agent *E. coli* GZ-34 as main active substances against *R. solanacearum*. We also revealed that cyclo(l-Pro-l-Phe) has antifungal activity as it could effectively inhibit spore formation of *Magnaporthe grisea* [22,23,24]. Our findings demonstrate that cyclic dipeptides from *E. coli* might be developed as new potential antimicrobial agents against both bacterial pathogens and fungal pathogens.

## 2. Results

### 2.1. Isolation of New Antagonistic Bacteria against R. solanacearum

**Figure 1:**
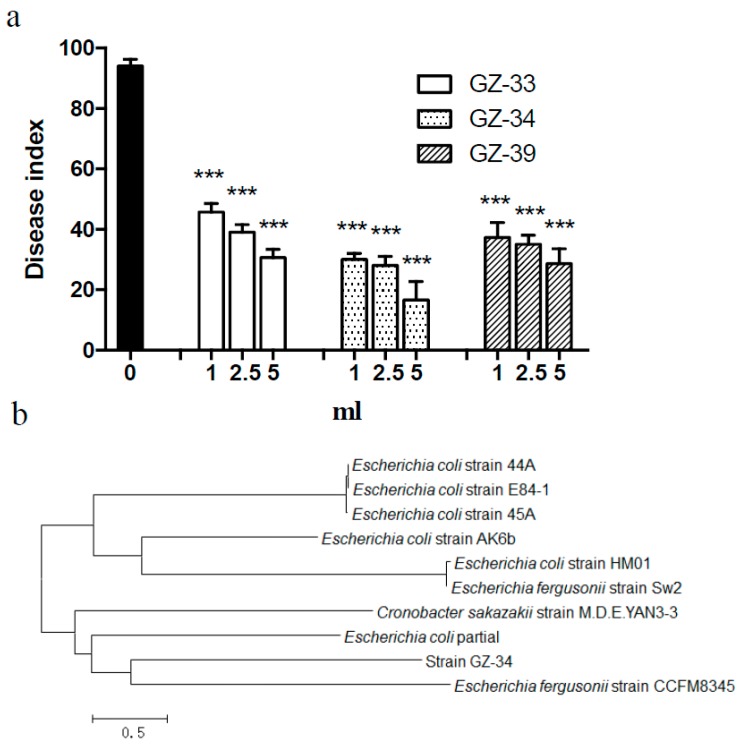
Isolation and identification of antagonistic bacteria. (**a**) The disease index of plant with treatment of the antagonistic bacteria of *Pantoea* GZ-33, *Escherichia* GZ-34, and *Dickeya* GZ-39 against *R. solanacearum*. *R. solanacearum* and the antagonistic bacteria were used at 1:0, 1:1, 1:2.5, and 1:5 (*v*/*v*). Data are means ± standard deviations from three independent experiments. *** *p* < 0.001 (unpaired *t*-test); (**b**) Analysis of phylogenic tree of *E. coli* GZ-34 based on 16S rDNA sequence. The 16S rDNA gene sequence was submitted to the NCBI website and is available with the supplied GenBank accession number MF374346.

To isolate new and effective biocontrol agents against *R. solanacearum*, we screened 30 soil samples collected from the different provinces in China. Among approximately 40,000 colonies, there were 56 isolates showing a high antagonistic activity against *R. solanacearum*. After analyzing the 16S rDNA sequences of these antagonistic bacterial strains, three bacterial species were identified as new biocontrol agents against *R. solanacearum*: *Pantoea* (GZ-33, CCTCC No. M 2016352) (Supplementary Figure S1a,d and Supplementary Table S1), *Escherichia* (GZ-34, CCTCC No. M 2016353) (Figure 1b, Supplementary Figure S1b, and Supplementary Table S1), and *Dickeya* (GZ-39, CCTCC No. M 2016354) (Supplementary Figure S1c,e and Table S1). In combination with analysis of 16S rDNA sequences, biochemical and physiological properties, strain GZ-34 was finally determined as *E. coli* (Figure 1 and Table 1).

### 2.2. E. coli GZ-34 Shows Effective Protection to Tomato from R. solanacearum Infection

To test the effective protection of the new antagonistic bacteria in host plant from *R. solanacearum*, we took *Pantoea* GZ-33, *Escherichia* GZ-34, and *Dickeya* GZ-39 as potential biocontrol agents to treat tomato plants. It was shown that treatment with the new antagonistic bacteria obviously inhibited wilt symptoms in tomato plants and increased the survival rate of the plants. In the absence of the antagonistic bacteria, wilt symptoms appeared in tomato from the 10th day after inoculation with *R. solanacearum*. Treatment with the antagonistic bacteria GZ-33, GZ-34, and GZ-39 (1:5, *v*/*v*) not only delayed wilt development but also reduced the disease index to 30.67, 16.67, and 28.67, respectively (Supplementary Figure S2 and Figure 1a). The relative control effects on *R. solanacearum* were 67.38%, 82.27%, and 69.50%, respectively. The bacterial strain GZ-34 was used for further investigation as it displayed an excellent antagonistic activity against *R. solanacearum*. 

### 2.3. E. coli GZ-34 Remarkably Inhibits the Cell Growth of R. solanacearum in Soil and Plants

**Figure 2:**
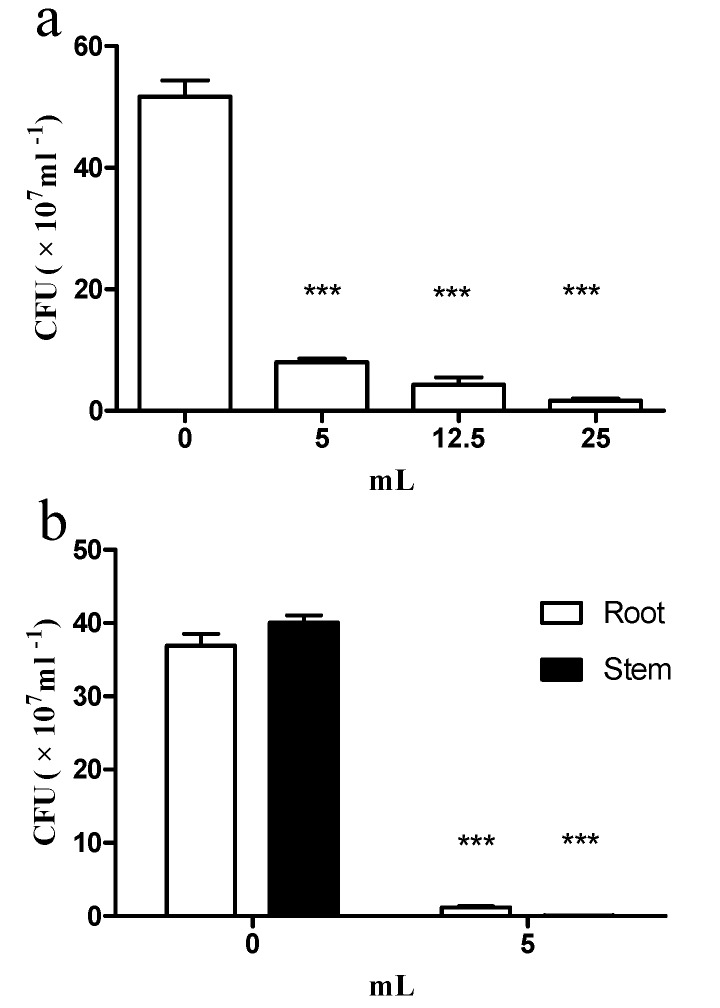
The inhibitory effect of *E. coli* GZ-34 on the cell growth of *R. solanacearum* in soil (**a**) and in plant (**b**). Data are means ± standard deviations from three independent experiments. *** *p* < 0.001 (unpaired *t*-test).

**Figure 3:**
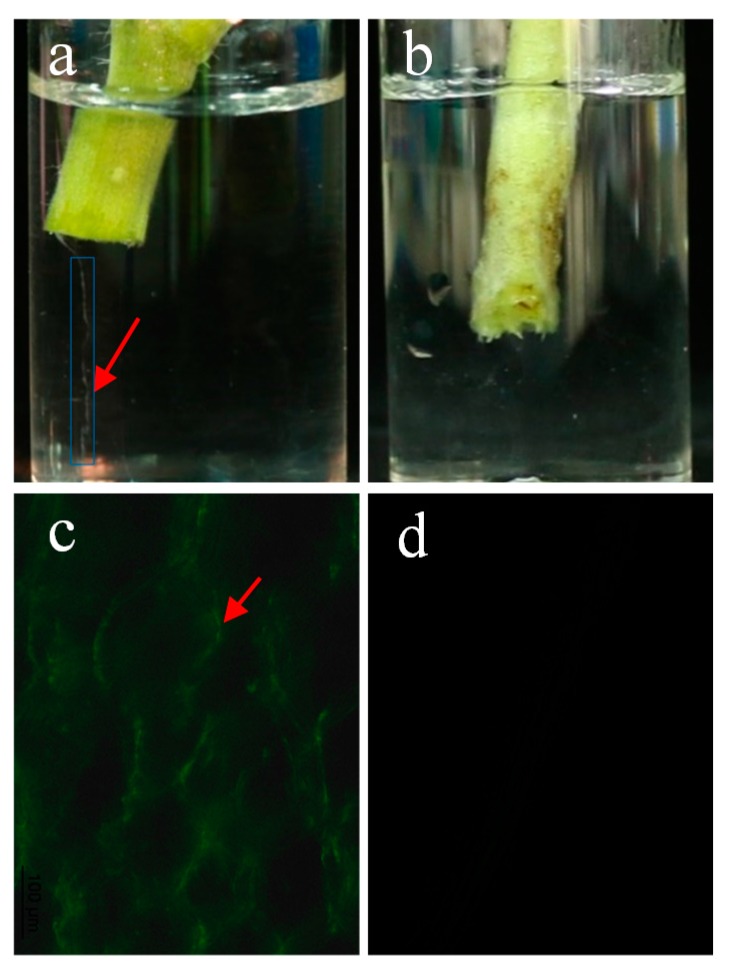
Influence of *E. coli* GZ-34 on *R. solanacearum* cell proliferation in the plant stem. After infection with *R. solanacearum*, the plant stem streamed bacterial milky liquid into sterile water without treatment with *E. coli* GZ-34 (**a**); while there was no milky liquid (**b**) when the plant was treated with *E. coli* GZ-34. Microscopy observation showed *R. solanacearum* cells proliferated in the plant stem in the absence of the biocontrol agent (**c**); but there was no detectable cells or signal in the plant in the presence of the biocontrol agent (**d**). Arrows pointed to *R. solanacearum* cells.

To further study the protective effect of *E. coli* GZ-34 on tomato plant, we measured the Colony-Forming Units (CFU) value of *R. solanacearum* in both soil and plants in the absence and presence of *E. coli* GZ-34. It was revealed that *R. solanacearum* cell numbers were strongly inhibited in the presence of *E. coli* GZ-34 (Figure 2). The CFU of *R. solanacearum* in soil decreased from 51.7 × 10^7^ to 1.7 × 10^7^ after treatment with 5 mL of *E. coli* GZ-34 culture (Figure 2a). A similar result was also observed in tomato roots and stems, the CFU was reduced by 96.75% and 99.87%, respectively, after addition of 5 mL *E. coli* GZ-34 culture (Figure 2b). It was also found that the stem of the tomato infected by *R. solanacearum* without treatment of *E. coli* GZ-34 streamed milky liquid into the sterile water (Figure 3a). This liquid represented the bacterial ooze exuding from the cut ends of colonized vascular bundles. There was no milky liquid from the plant stem when it was treated with *E. coli* GZ-34 (Figure 3b). Microscopy observation also supported similar results (Figure 3c,d).

### 2.4. Structural Characterization of Antimicrobial Compounds Isolated from E. coli GZ-34

**Figure 4:**
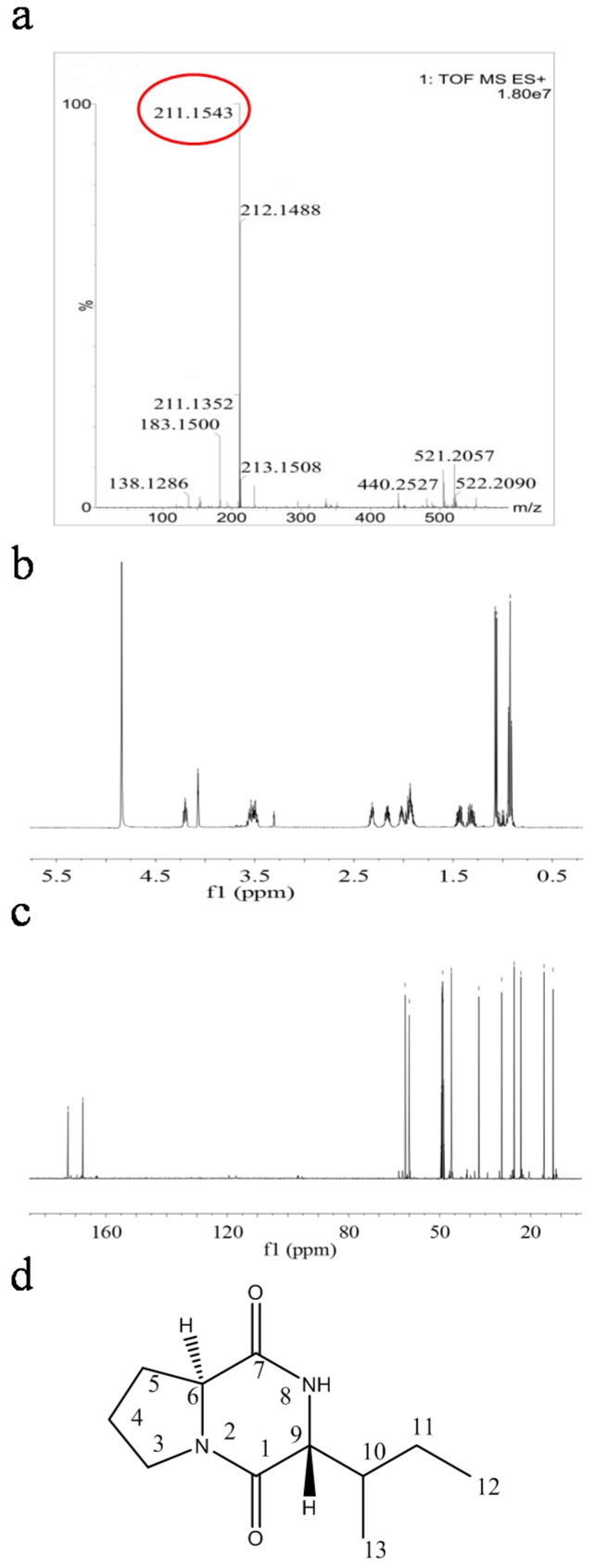
Structural characterization of fraction 1. (**a**) ESI-MS spectra of fraction 1; (**b**) ^1^H-NMR spectra of fraction 1; (**c**) ^13^C-NMR spectra of fraction 1; (**d**) Structure of fraction 1 was identified as cyclo(l-Pro-d-Ile).

To determine the chemical structures of antimicrobial compounds from *E. coli* GZ-34, the active components were isolated and purified from 70 L culture supernatant. Two fractions showed a strong antimicrobial activity against *R. solanacearum.* Approximately 43.1 mg and 80.6 mg of the two fractions were obtained by HPLC separation. The purity of fractions 1 and 2 were 95% and 97%, respectively. Fraction 1 was identified as a dipeptide based on the presence of both the characteristic ^13^C-NMR chemical shifts for the amide carbonyl groups at *δ*_C_ 167.7-172.6 and the typical ^1^H-NMR signals for amino acids. The presence of a proline moiety of fraction 1 was deduced from the presence of CH_2_ multiplets in the spectra at *δ*_H_ 3.60-3.53 (1H, m), 3.52-3.49 (1H, m), 2.06-2.02 (1H, m), 1.98-1.91(1H, m), 2.35-2.32 (1H, m), 2.00-1.92 (1H, m). The ^1^H-NMR spectrum showed a γ-methyl doublet at *δ*_H_ 0.95 (3H,d, *J* = 6.9 HZ, Me(13)), and a terminal Me triplet at *δ*_H_ 1.07 (3H, t, *J* = 7.1 HZ, Me(12)), indicating the presence of an anteiso branched chain. In addition, the signals at *δ*_H_ 4.22 (1H, t, *J* = 7.1 HZ, HC(9)), 2.20-2.16 (1H, m, HC(10)), and 1.49-1.43 (m, 1H of CH_2_(11)), 1.37-1.31 (m, 1H of CH_2_(11)) allowed to propose an Ile moiety for this compound. Furthermore, high-resolution electrospray ionization mass spectrometry analysis of the fraction 1 revealed a molecular ion [M + H]^+^ with an *m*/*z* of 211.1543, suggesting a molecular formula of C_11_H_18_N_2_O_2_. ^1^H- and ^13^C-NMR previously reported data for fraction 1 are in agreement with the obtained data [25], which indicate that fraction 1 is cyclo(l-Pro-d-Ile) (Figure 4, Supplementary Figure S3 and Supplementary Table S2).

**Figure 5:**
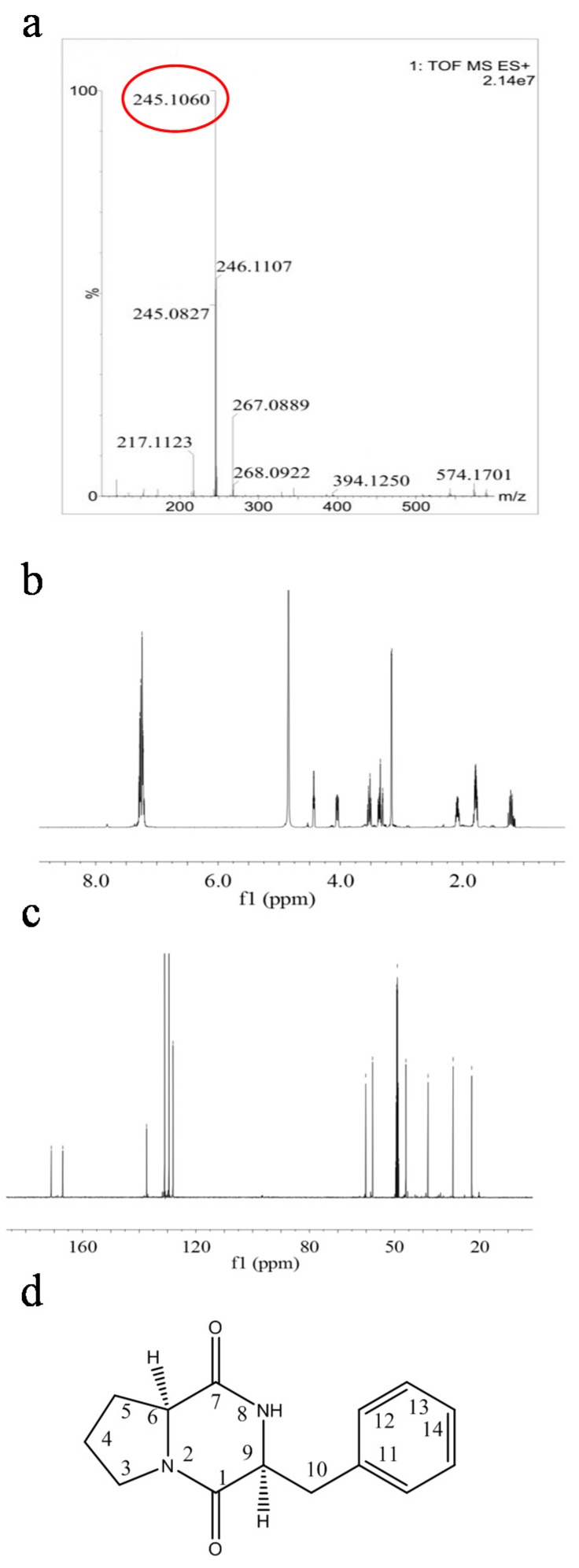
Structural characterization of fraction 2. (**a**) ESI-MS spectra of fraction 2; (**b**) ^1^H-NMR spectral of fraction 2; (**c**) ^13^C-NMR spectra of fraction 2; (**d**) The structure of fraction 2 was identified as cyclo(l-Pro-l-Phe).

The identification of fraction 2 as a dipeptide was straight forward based on the presence of both the characteristic ^13^C-NMR chemical shifts for the amide carbonyl groups at *δ*_C_ 166.9-171.1 and the typical ^1^H-NMR signals of amino acids. The presence of a proline moiety in fraction 2 was deduced by the presence of CH_2_ multiplets in the spectra at *δ*_H_ 3.56-3.48 (1H, m), 3.39-3.33 (1H, m), 1.84-1.75 (2H, m), 1.24-1.15 (1H, m), and 2.12-2.04 (1H, m). The ^1^H-NMR spectra of fraction 2 displayed signals of methylene benzylic protonsgroups (H-10) attached to monosubstituted benzene rings (*δ*_H_ 3.14-3.18). These data suggested a phenylalanine moiety for these compounds. High-resolution electrospray ionization mass spectrometry analysis of fraction 2 revealed a molecular ion [M + H]^+^ with an *m*/*z* of 245.1060, suggesting a molecular formula of C_14_H_16_N_2_O_2_. The previously-reported ^1^H- and ^13^C-NMR data for fraction 2 are in agreement with the obtained data [22,26,27], which indicate that fraction 2 is cyclo(l-Pro-l-Phe) (Figure 5, Supplementary Figure S4 and Supplementary Table S2).

### 2.5. Antimicrobial Compounds Isolated from E. coli GZ-34 Interfere with Cell Growth and Expression Levels of Virulence Contributors of R. solanacearum

**Figure 6:**
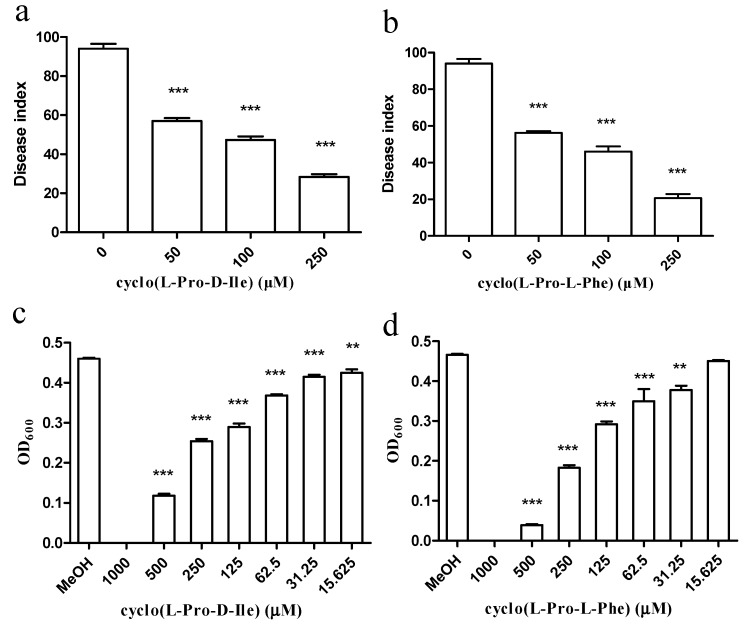
Influence of cyclo(l-Pro-d-Ile) and cyclo(l-Pro-l-Phe) on *R. solanacearum.* The disease index of the plant infected by *R. solanacearum* with treatment of cyclo(l-Pro-d-Ile) (**a**) and cyclo(l-Pro-l-Phe) (**b**); antimicrobial activity of cyclo(l-Pro-d-Ile) (**c**) and cyclo(l-Pro-l-Phe) (**d**) against *R. solanacearum* were measured. Data are means ± standard deviations from three independent experiments. ** *p* < 0.01; *** *p* < 0.001 (unpaired *t*-test).

The purified antimicrobial compounds from *E. coli* GZ-34 obviously inhibited wilt symptoms in tomato plants and increased the survival rate of the plants (Figure 6a,b). To fully learn the inhibitory effect of cyclo(l-Pro-d-Ile) and cyclo(l-Pro-l-Phe) on *R. solanacearum*, the MIC values of the two antimicrobial compounds were measured and determined as 1000 μM (Figure 6c,d). We continued to study whether the two compounds affect the virulence contributors in *R. solanacearum*. Transcriptional expression levels of the genes related to extracellular polysaccharides, motility, T3SS, TTE, T4SS pili, chemotaxis, and the global regulator were tested in the presence of the compounds at 100 μM using the primers listed in Table S3. It was revealed that exogenous addition of cyclo(l-Pro-d-Ile) and cyclo(l-Pro-l-Phe) downregulated the expression levels of *hrpB*, *pil*Q and cellulase encoding gene. HrpB is a master regulator to be involved in regulation of T3SS, chemotaxis and biosynthesis of various low molecular weight compounds. Addition of cyclo-(l-Pro-l-Phe) also decreased many other gene expressions, such as *phcA*, *epsF*, *fliT*, *pilQ*, and *cheW*. PhcA controls extracellular polysaccharides, cellulose, and AHL quorum sensing system.

### 2.6. Cyclo(l-Pro-l-Phe) Inhibits Spore Formation in M. Grisea

**Figure 7:**
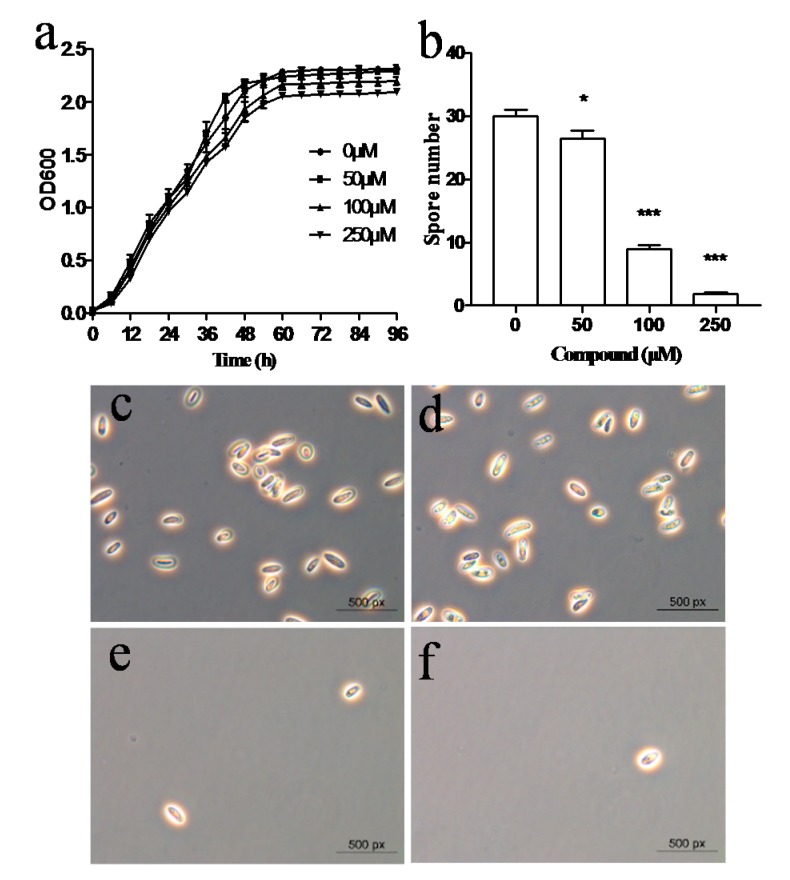
Effect of cyclo(l-Pro-l-Phe) on the spore formation of *M. grisea*. Growth curve (**a**) and spore formation (**b**) of *M. grisea* in the presence of different final concentrations of cyclo(l-Pro-l-Phe) as indicated. Microscopic analysis of spore formation of *M. grisea* with addition of cyclo(l-Pro-l-Phe) at a final concentration of 0 (**c**); 50 μM (**d**); 100 μM (**e**); and 250 μM (**f**). Data are means ± standard deviations from three independent experiments. * *p* < 0.05; *** *p* < 0.001 (unpaired *t*-test).

To determine whether the biocontrol agent *E. coli* GZ-34 has antifungal activity, we selected several important fungal pathogens and found that the ethyl acetate extract of *E. coli* GZ-34 could inhibit spore formation in *M. grisea*, which is the causal agent of rice blast. The subsequent study revealed that the active compound was cyclo(l-Pro-l-Phe) from *E. coli* GZ-34 (Figure 7). The spore formation ratio of *M. grisea* decreased from 100% to 70.45% 29.49%, and 6.44% in the presence of 50 μM, 100 μM, and 250 μM of cyclo(l-Pro-l-Phe), respectively, suggesting that cyclo(l-Pro-l-Phe) is a key component responsible for the inhibitory activity of *E. coli* GZ-34 on the spore formation of *M. grisea*. Interestingly, we found that the ethyl acetate extract of *E. coli* GZ-34 could also inhibit both the growth and sexual integration of *Sporisorium scitamineum* (Supplementary Figure S5), suggesting that *E. coli* GZ-34 has a broad-spectrum antifungal activity. However, the two compounds only showed a slight effect on *S. scitamineum*.

## 3. Discussion

Biological control shows an obvious advantage over traditional methods, such as chemical control and the development of resistant cultivars. Many microorganisms have been identified for their antimicrobial activity against plant pathogens. In addition, phage was also extensively explored to control plant pathogens [28]. However, limitations of phage therapy, such as phage adsorption blocked by pathogenic bacteria extracellular polysaccharide, and the various degrees of susceptibility among many bacterial strains, inhibit the wide application of this method. In this study, we have isolated 56 biocontrol agents with high antagonistic activity against *R. solanacearum*. Our results showed that there are three new antagonistic bacterial species identified as *Pantoea*, *Escherichia*, and *Dickeya*. These new biocontrol agents display effective antimicrobial activity, and could protect plants from infection by *R. solanacearum* (Figure 1, Figure 2 and Figure 3 and Figure 6). Our study provides one more evidence to apply biocontrol agents to control bacterial wilt caused by *R. solanacearum*, which is the causative agent of bacterial wilt in many important crops worldwide.

Characterization of the isolated biocontrol agents showed that *E. coli* GZ-34 had a high antimicrobial activity against *R. solanacearum.* Pot experiments demonstrated that the application of *E. coli* GZ-34 could reduce disease index of plant to 16.7% (Figure 1). Lugtenberg et al. [29] reported that competitive root tip colonization by *Pseudomonas* strains can play an important role in the control of soil-borne crop diseases caused by fungi. Inadequate colonization is often the limiting factor for biological control, but *E. coli* GZ-34 showed a good efficacy in disease suppression in the greenhouse. Another advantage of *E. coli* GZ-34 is the broad-spectrum activity against pathogens. The ethyl acetate extract of *E. coli* GZ-34 inhibited both the growth and sexual integration of *S. scitamineum*, which is an important fungal pathogen of sugarcane (Supplementary Figure S5) [30]. Cyclo(l-Pro-l-Phe) produced by *E. coli* GZ-34 could effectively reduce spore formation of *M. grisea* (Figure 7)*. M. grisea* is the causal agent of rice blast, and spore formation is an important process for its pathogenesis. These results demonstrate that *E. coli* GZ-34 is a promising potential biocontrol agent against both bacterial and fungal pathogens.

Several types of cyclic dipeptides have been identified for their antimicrobial activities [31,32,33,34], indicating that they would be potential for antibiotic design. Some dipeptides were also reported to reduce virulence-factor production in bacterial pathogens [33,35,36]. Our study demonstrate the vital role of cyclic dipeptide in control of bacterial pathogens. Both cyclo(l-Pro-d-Ile) and cyclo(l-Pro-l-Phe) showed an antibacterial activity (Figure 6). Cyclo(l-Pro-l-Phe) also showed a strong antifungal activity against *M. grisea* to inhibit the spore formation (Figure 7)*.* Interestingly, cyclo(l-Pro-d-Ile) and cyclo(l-Pro-l-Phe) may interfere with the production of some virulence contributors in *R. solanacearum* (Table S4). These findings suggest that cyclic dipeptides could be developed as novel antimicrobial compounds not only to inhibit the cell growth of pathogens, but also to interfere with the virulence-regulating systems in pathogens. 

## 4. Materials and Methods

### 4.1. Bacterial and Fungal Growth Conditions and MIC Assays

The bacterial strains and plasmids used in this work are listed in Table S1. *R. solanacearum* GMI1000(ATCCBAA-1114) was obtained from the American Type Culture Collection (ATCC) and maintained at 28 °C in 2,3,5-triphenyltetrazolium chloride medium (TTC medium) consisting of 1% tryptone, 0.1% casamino acids, 5% glucose, and 0.005% 2,3,5-triphenyltetrazolium chloride. The GMI1000(egfp) strain harboring the expression construct pBBR1-2-eGFP was constructed in this study. Luria−Bertani (LB) medium (1 liter contains 10 g tryptone, 5 g yeast extract, and 10 g NaCl, pH 7.0) was used to isolate biocontrol agents. *Magnaporthe grisea* Guy11(ATCC201236) was cultured in rice bran culture medium (20 g rice bran, 2 g yeast extract, and 11 g agar per 1 liter). *Sporisorium scitamineum* was cultured in YePSA medium (10 g yeast extract, 20 g peptone, 20 g sugar, and 20 g agar per liter) [30]. The antibiotic kanamycin (100 μg/mL) was used when necessary. The minimum inhibitory concentration (MIC) was defined as the lowest concentration of antimicrobial compounds in which bacterial growth in the well was not measurable by determining the turbidity at 600 nm, following the method from the Clinical and Laboratory Standards Institute (CLSI) [37].

### 4.2. Isolation of Antagonistic Bacteria against R. solanacearum

The soil samples (10 g) were dissolved in 90 mL of sterilized water, and the suspensions were serially diluted and spread on King’s B agar [38]. After incubating at 30 °C for two days, single bacterial colonies were selected and streaked onto a new LB plate. Antagonistic activities were evaluated by measuring the diameter of inhibition zones of antagonistic bacteria in the bioassay plate of *R. solanacearum* (10^6^ CFU/mL) after incubation at 28 °C for three days. 

### 4.3. Characterization of Antagonistic Bacteria against R. solanacearum

Biochemical and physiological properties of bacterial antagonists were determined by analyzing the Gram reaction, oxidase activity, starch hydrolysis, arginine dihydrolase, V-P detection, nitrate reduction, and growth at different temperatures according to standard methods [39]. They were also characterized by partial nucleotide sequencing of 16S rDNA gene using PCR with 27-f (AGAGTTTGATCCTGGCTCAG) and 1492-r (GGTTACCTTGTTACGACTT) primers [40]. Nucleotide sequences were aligned using ClustalW available in the molecular evolutionary genetics analysis (MEGA) 5.2 program (Molecular Biology and Evolution, New York, NY, USA) [41]. The 16S rDNA gene sequence was submitted to the National Center for Biotechnology Information database (NCBI) and is available with the supplied GenBank accession number MF374346. The three potential biocontrol agents were also submitted to the China Center for Culture Collection (CCTCC). The collection numbers re provided in Table S1.

### 4.4. Construction of GMI1000(eGFP) Strain

The eGFP coding region was amplified using the primers of egfp-F and egfp-R and PET-28a-eGFP as a template. The PCR product was digested with XhoI and HindIII and ligated into the same enzyme sites of pBBR1-2 for generating the construct pBBR1-2-eGFP. The construct was verified by DNA sequencing before it was introduced into GMI1000 by electroporation. The GMI1000 transconjugants were selected on TTC plates containing kanamycin.

### 4.5. Analysis of Efficacy against Tomato Bacterial Wilt

Efficacy of antagonistic bacteria against tomato bacterial wilt was proceeded in greenhouse AIRKINS (28 °C, 16 h (light) plus 8 h (dark)). A mixture of field soil, sand and compost (1.25:1.25:0.5) was prepared and autoclaved at 121 °C for 20 min. The plants were inoculated with 5 mL of *R. solanacearum* GMI1000(eGFP) culture at an OD_600_ = 3.0 supplemented with biocontrol agents at 1:0, 1:1, 1:2.5, and 1:5 (*v*/*v*), respectively. The biocontrol agents were also grown to OD_600_ = 3.0. TTC medium and LB medium were used in the blank control group. Biocontrol agent cells were grown in LB medium and GMI1000 (eGFP) cells were grown in TTC medium. 

Tomato seeds (Jinfeng 1) were treated by 2% NaClO for 3 min and 75% ethanol for 2 min. The seeds were rinsed with sterile water three times before being planted for 30 d. Each treatment had 25 replicates. The plants were scored for disease index (DI) on a scale of 0–4 (Table 2) [42]:

$$\mathrm{Disease}\;\mathrm{index}(\mathrm{DI})=\frac{\sum \left(\mathrm{The}\;\mathrm{number}\;\mathrm{of}\;\mathrm{diseased}\;\mathrm{plants}\;\mathrm{in}\;\mathrm{each}\;\mathrm{grade}\times \mathrm{grade}\right)}{\mathrm{Total}\;\mathrm{number}\;\mathrm{of}\;\mathrm{plants}\;\mathrm{investigated}\times \mathrm{The}\;\mathrm{highest}\;\mathrm{disease}\;\mathrm{index}}\times 100\%$$

$$\mathrm{Control}\;\mathrm{efficacy}\;(\%)=\frac{\mathrm{DI}\;\mathrm{in}\;\mathrm{GMI}1000\;\mathrm{group}\;-\;\mathrm{DI}\;\mathrm{in}\;\mathrm{the}\;\mathrm{antagonist}\;\mathrm{treated}\;\mathrm{group}}{\mathrm{DI}\;\mathrm{in}\;\mathrm{GMI}1000\;\mathrm{group}\;}\times 100\%$$

Data were analyzed for significance using analysis of variance followed by Ducan’s least-significant-difference test (*p* = 0.05), using the SAS software program. All experiments were performed at least three times.

### 4.6. Analysis of R. solanacearum Cell Numbers in Soil and in Plants

One gram of soil, plant root, and stem were collected and milled in 9 mL of sterilized water for 20 min, respectively. The suspension was then diluted and spread on TTC plates. eGFP-expressing colonies (GFP-CFU) were enumerated using an epifluorescence binocular microscope (M165 FC, Leica, Leica Microsystems, Germany) at low magnification (40×), whereas colonies on plates were counted without magnification under normal light. The microscopic analysis of *R. solanacearum* cells in plant stems were prepared using two methods. First, the plant stems were thinly sliced and placed in water, and the bacterial cells were flowed to accumulate in the bottom [43]. Second, the thinnest stem slice was picked up and placed on a glass slides, and was covered for a fluorescence microscope assay (DMi8, Leica, Leica Microsystems, Germany).

### 4.7. Analysis of Antimicrobial Compounds

*E. coli* GZ-34 cells were cultured in LB medium overnight and the supernatant was extracted with an equal volume of ethyl acetate. Following evaporation of the ethyl acetate, the residue was dissolved in methanol and subjected to HPLC analysis on a C18 reverse phase column (XBridge, 10 μm, 19 mm × 250 mm, Waters) and eluted with methanol-water (from 10:90 to 100:0 *v*/*v*) at a flow rate of 7 mL/min for 30 minutes. Elution was collected for the test of antimicrobial activity. The active fraction 1 was detected and purified by HPLC using a semi-preparative C18 reverse phase column (Gemini-NX, 50 μm, 10.0 mm × 250 mm, Phenomenex) and eluted with methanol-water (10:90 *v*/*v*) at a flow rate of 3 mL/min. The active fraction 2 was detected and purified by HPLC using a semi-preparative C18 reverse phase column (Gemini-NX, 50 μm, 10.0 mm × 250 mm, Phenomenex) and eluted with methanol-water (33:67 *v*/*v*) at a flow rate of 3 mL/min. Peaks were monitored using a UV detector at 210 nm and were collected and assayed. The purities of fractions 1 and 2 were 95% and 97%, respectively.

The ^1^H, ^13^C, and DEPT135 nuclear magnetic resonance (NMR) spectra in CD_3_OD solution were obtained using a Bruker AV-500 (Bruker Instrument, Inc., Zurich, Switzerland) spectrometer operating at 500 MHz for ^1^H or 125 MHz for ^13^C. High-resolution electrospray ionization mass spectrometry was performed on a Waters Q-Tof Premier high-resolution mass spectrometer (Waters, USA, MA).

### 4.8. Microscopic Analysis and Quantification of Spore Formation in M. grisea

*M. grisea* is a typical heterothallic ascomycete and the causal agent of rice blast, one of the most destructive diseases of rice worldwide. Spore formation is important for their infection and transmission [44,45]. For testing the effect on spore formation in *M. grisea*, cyclo(l-Pro-l-Phe) was diluted in methanol and added at the final concentrations as indicated. *M. grisea* was cultured in rice bran culture medium at 28 °C for five days. Three milliliters of sterile water was used to scrape the mycelium with a sterile coated rod. The cultures were incubated at 28 °C for five days under 8-h light plus 16-h dark conditions. Spores was washed using 5 mL of sterile water and quantified using a phase contrast microscope (DMi8, Leica, Germany). Imaging was achieved using a Leica DMI8 fluorescence microscope with DIC 100× and a Leica DFC 450C camera interphased with LAS V4.8 software. 

## Figures and Tables

**Table 1:** Morphological, physiological, and biochemical characteristics of *E. coli* GZ-34.

Test	Result	Test	Result
Cell morphology	Long rod	Gram stain	-
Oxidase	-	Contact enzyme	+
Citrate	-	V-P determination	-
Hydrogen sulfide	-	Lysine decarboxylase	-
Ornithine decarboxylase	-	Arginine dihydrolase	-
Nitrate	+	Starch hydrolysis	-
Urease	-	Lecithinase	-
Indole test	+	Cellobiose	-
Arabinose	-	Lactose	-
Sorbitol	+	Adonitol	-
Glucose	+	Maltose	+
Xylose	+	Trehalose	+
Mannitol	+	ONPG	-
Malonate	-	Methyl red	+

Note: + denotes Positive, - denotes Negative.

**Table 2:** Grading criteria of severity of *R. solanacearum.*

Grade	Disease Description
DI-0	No visible symptoms
DI-1	Up to 25% of leaves wilted
DI-2	25–50% of leaves wilted
DI-3	50–75% of leaves wilted
DI-4	75–100% of leaves wilted, the plants always died

## Supplementary Materials

The following are available online, Figure S1: Isolation and characterization of antagonistic bacteria, Figure S2: Effect of antagonistic bacteria on tomato infected by *R. solanacearum*, Figure S3: DEPT135 spectra of cyclo(l-Pro-d-Ile), Figure S4: DEPT135 spectra of cyclo(l-Pro-l-Phe), Figure S5: Effect of the ethyl acetate extract of GZ-34 on the sexual integration of *S. scitamineum*, Table S1: Bacterial strains and plasmids used in this study, Table S2: ^1^H (500 MHz) and ^13^C (125 MHz) NMR data of fraction 1 and fraction 2 (δ in ppm),Table S3: Primers used in this study, Table S4: Transcriptional expression levels of virulence-related genes in *R. solanacearum* after treatment with antimicrobial compounds from *E. coli* GZ-34.
