# Supplementary material for: Identification of Cyclic Dipeptides from Escherichia coli as New Antimicrobial Agents against Ralstonia Solanacearum
Source: Molecules. 2018 Jan 19;23(1):214. doi: 10.3390/molecules23010214 (PMC6017746; doi:10.3390/molecules23010214)
Supplement: Supplementary file 1 [file molecules-23-00214-s001.pdf]

Supplementary Materials:

Identification of cyclic dipeptides from *Escherichia coli* as new antimicrobial agents against *Ralstonia solanacearum*

Shihao Song[1,2#], Shuna Fu[1,2,3#], Xiuyun Sun[1,2,3#], Peng Li[4], Ji'en Wu[5], Tingyan Dong[1,2], Fei He[3], and Yinyue Deng[1,2,3]*

[1]*State Key Laboratory for Conservation and Utilization of Subtropical Agro-Bioresources, South China Agricultural University, Guangzhou 510642, China*

[2]*Guangdong Innovative Research Team of Sociomicrobiology, College of Agriculture, South China Agricultural University, Guangzhou 510642, China*

[3]*Integrative Microbiology Research Centre, South China Agricultural University, Guangzhou 510642, China*

[4]*School of Biological and Science Technology, University of Jinan, Jinan 250022, China;*

[5]*Department of Chemistry, National University of Singapore, Science Drive 3, Singapore, 117543, Singapore*

[#]These authors have contributed equally to this work.

***Corresponding author:**

Yinyue Deng:

College of Agriculture, South China Agricultural University

Tel: 86-20-85286471;

Email address: ydeng@scau.edu.cn

1. Experimental methods:

1.1 Quantitative Real-Time PCR Assays.

R. solanacearum cells were cultured and harvested till OD_{600}=1.0. RNA was isolated using the RNeasy Protect Bacteria Mini Kit (Qiagen, Copenhagen, Denmark), and was treated using the Turbo DNA-free kit (Ambion, Life Technologies, Denmark) according to manufacturers' instructions. cDNA synthesis and quantitative RT-PCR analysis were carried out using the Qscript 1-Step Sybr green qRT-PCR kit (Quanta Biosciences, Gaithersburg, MD) according to manufacturer's instructions. Using 7300Plus Real-Time PCR System. As a control, quantitative RT-PCR was similarly applied to analyze the expression of the 16S rRNA gene. The relative expression levels of the target genes were calculated using the Quantitation-Comparative CT (ΔΔCT) method [1].

1.2 Effect of ethyl acetate extract of *E. coli* GZ-34 on *Sporisorium scitamineum.*

S. scitamineum MAT-1 and MAT-2 were cultured in YePSA medium and harvested till OD_{600}=1.5. MAT-1 and MAT-2 were mixed with equal volumes and 1 μl mixtures were pointed on the YePSA solid medium plates supplementing with the ethyl acetate extract of *E. coli* GZ-34 as indicated. After being cultured at 28 °C for 3 days, the colonies were observed using a stereomicroscope (M165 FC, Leika, Germany) at low magnification (10x) [2].

References

1. Kovach ME, Elzer PH, Hill DS, Robertson GT, Farris MA, Roop RM 2nd, Peterson KM. Four new derivatives of the broad-host-range cloning vector pBBR1MCS, carrying different antibiotic-resistance cassettes. *Gene* **1995**, 166:175-176, DOI:10.1016/0378-1119(95)00584-1.

2. Yan M, Zhu G, Lin S, Xian X, Chang C, Xi P, Shen W, Huang W, Cai E, Jiang Z, Deng YZ, Zhang LH. The mating-type locus b of the sugarcane smut *Sporisorium scitamineum* is essential for mating, filamentous growth and pathogenicity. *Fungal Genet Biol* **2016**, 86:1-8, DOI:10.1016/j.fgb.2015.11.005.

Table S1. Bacterial strains and plasmids used in this study

Strain or plasmid	Phenotype and/or characteristic(s)	Source or reference
Strain		
GMI1000	Wild-type strain of *R. solanacearum*	ATCCBAA-1114
GMI1000-eGFP	GMI1000 containing the egfp gene	This study
GZ-33	An antagonistic bacterium against GMI1000	CCTCC NO: M 2016352
GZ-34	An antagonistic bacterium against GMI1000	CCTCC NO: M 2016353

GZ-39	An antagonistic bacterium against GMI1000	CCTCC NO: M 2016354
Guy11	*M. grisea*	ATCC201236
Ss17 (MAT-1)	Pair of mating strains of *S. scitamineum*	Yan et al., 2016
Ss18(MAT-2)	Pair of mating strains of *S. scitamineum*	Yan et al., 2016
Plasmid		
pBBR1MCS-2	Broad-host-range cloning vector, Km^r	Kovach et al. (1995)
pBBR1-eGFP	pBBR1MCS-2 containing the egfp gene	This study

Table S2. ^{1}H (500 MHz) and ^{13}C (125 MHz) NMR data of fraction 1 and fraction 2 (δ in ppm)

Position	**Fraction 1** (in CD_3OD)		**Fraction 2** (in CD_3OD)	
	δ (H)	δ (C)	δ (H)	δ (C)
1		172.6		166.9
2				

3	3.60-3.53(1H,m),<br>3.52-3.49(1H,m)	46.3	3.56-3.48(1H,m),<br>3.39-3.33(1H,m)	46.1
4	2.06-2.02(1H,m),<br>1.98-1.91(1H,m)	23.4	1.84-1.75 (2H, m)	22.8
5	2.35-2.32(1H,m),<br>2.00-1.92(1H,m)	29.7	1.24-1.15(1H,m),<br>2.12-2.04(1H,m)	29.4
6	4.10(1H,br,s)	61.4	4.06(1H, ddd, *J* =<br>1.7,6.3,10.8 HZ)	60.2
7		167.7		171.1
8			4.56(1H,br,s)	
9	4.22(1H,t,*J* = 7.1 HZ)	60.1	4.44(1H, ddd, *J* = 1.0,4.8,5.0 HZ)	57.8
10	2.20-2.16(1H,m)	25.6	3.18(1H, dd, *J* = 4.8,14.4 HZ),<br>3.14(1H, dd, *J* = 5.0,14.4 HZ)	38.2
11	1.49-1.43(1H,m),<br>1.37-1.31(1H,m)	37.2		137.5
12	1.07(3H,t,*J* = 7.1 HZ)	15.7	7.29-7.20(1H,m)	131.2
13	0.95 (3H,d, *J* = 6.9 HZ)	12.7	7.29-7.20(1H,m)	129.6
14			7.29-7.20(1H,m)	128.2

Table S3. Primers used in this study

Primer name	Prime sequence	Note
16s-27f	AGAGTTTGATCCTGGCTCAG	
16s-1492r	GGTTACCTTGTTACGACTT	
egfp-F	CCGCTCGAGATGGTGAGCAAGGGCGAGGAG	*Xho*I
egfp-R	CCCAAGCTTTCAAAGATCTACCATGTACAGCTCGT	*Hind*III
epsA-F	GTACCGAAGATCACGCCCAT	qRT-PCR
epsA-R	GATCCCAGACCACGATCGAC	qRT-PCR
epsE-F	GGCAAGTTCTGGCGCAATTT	qRT-PCR
epsE-R	CGTCTGGAACAGGATCAGGC	qRT-PCR
epsF-F	TGCGTTCTACGAGTTCCAGC	qRT-PCR
epsF-R	TTGGCCACGGAATACGAGAG	qRT-PCR
motA-F	GCTAGTCGCCATCGGTTACA	qRT-PCR
motA-R	GATCGCCTTCTTGTCGTTGC	qRT-PCR
fliT-F	CAACTGGGAAGTCGTCAGCA	qRT-PCR
fliT-R	CGCGCATCGTCTTCGAGAAT	qRT-PCR
hrpB-F	TGCAGACCAAGGTGGAAGTC	qRT-PCR
hrpB-R	GAAGTCGAAATTCCAGCGGC	qRT-PCR
awr-F	GACAAGCGTTACAAGAGCGG	qRT-PCR
awr-R	GACCTTGAACTCCTGCTCGG	qRT-PCR
pilQ-F	GCCTGAGCGTCATCTTCGAT	qRT-PCR
pilQ-R	ATGTCGACCGTGTCTTCGAG	qRT-PCR
chew-F	CGAGGAATACGGCATCGACA	qRT-PCR
chew-R	GACGACGGTGTACTGGTTGT	qRT-PCR
phcA-F	CTTCAACATCAGCTTCGCCG	qRT-PCR
phcA-R	TCCAGCTCATTGGAACGCAT	qRT-PCR

G16S-F	CGATGTCTGCCTGTTCGACG	qRT-PCR
G16S-R	AGCCAGTCCATCTTGTCGC	qRT-PCR
cel-F	CTGCTCGATCCGCACAACTA	qRT-PCR
cel-R	ATTGCCCTTGAACTGGGTGG	qRT-PCR

Table S4. Transcriptional expression levels of virulence-related genes in *R. solanacearum* after treatment with antimicrobial compounds from *E. coli* GZ-34.

Gene name or ID	Fold changes compared to untreated		Description
	cyclo(L-Pro-D-Ile)	cyclo(L-Pro-L-Phe)	
RS22045 *epsA*	1.087±0. 136	0.944±0. 209	EPS I polysaccharide export outer membrane protein EpsA
RS22020 *epsE*	12.113**±4. 335	6.598**±2. 732	EPS I polysaccharide export inner membrane protein EpsE
RS22015 *epsF*	1.637**±0. 213	0.274***±0. 125	EPS I polysaccharide export inner membrane protein EpsF
motA	1.204±0. 198	0.674*±0. 167	Flagellar motor stator protein MotA

RS19010 *fliT*	0.869±0. 231	0.483*±0. 146	Flagellar protein FliT
RS21310 *hrpB*	0.648**±0. 108	0.641*±0. 139	Regulatory protein HrpB
RS21180 *awr*	0.79±0. 129	1.528*±0. 302	AWR family protein
RS11555 *pilQ*	0.695±0. 142	0.656±0. 204	GSPD
RS23915 *cheW*	1.066±0. 113	0.59**±0. 184	Chemotaxis protein CheW
RS13750 *phcA*	1.529**±0. 225	0.533*±0. 173	Transcriptional regulator
RS17915 *cel*	0.755*±0. 126	0.716*±0. 133	Drug: proton antiporter

Data are means ± standard deviations from three independent experiments. *, $P < 0.05$; **, $P < 0.01$; ***, $P < 0.001$ (unpaired t test).

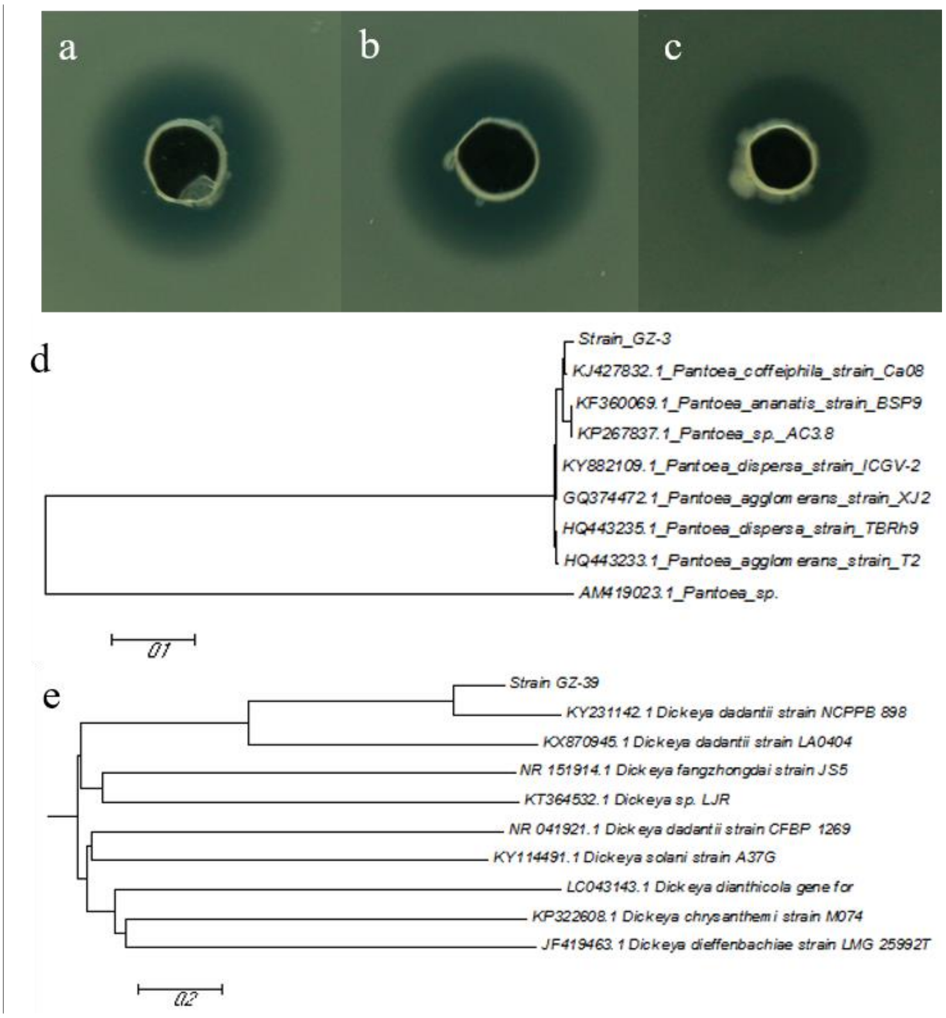

Fig. S1. Isolation and characterization of antagonistic bacteria. The inhibition zone of GZ-33 (a), GZ-34 (b) and GZ-39 (c) in *R. solanacearum* bioassay plate. Analysis of phylogenic tree of GZ-33 (d) and GZ-39 (e), which was based on 16S rRNA sequences. Each experiment was performed at least three times in triplicate.

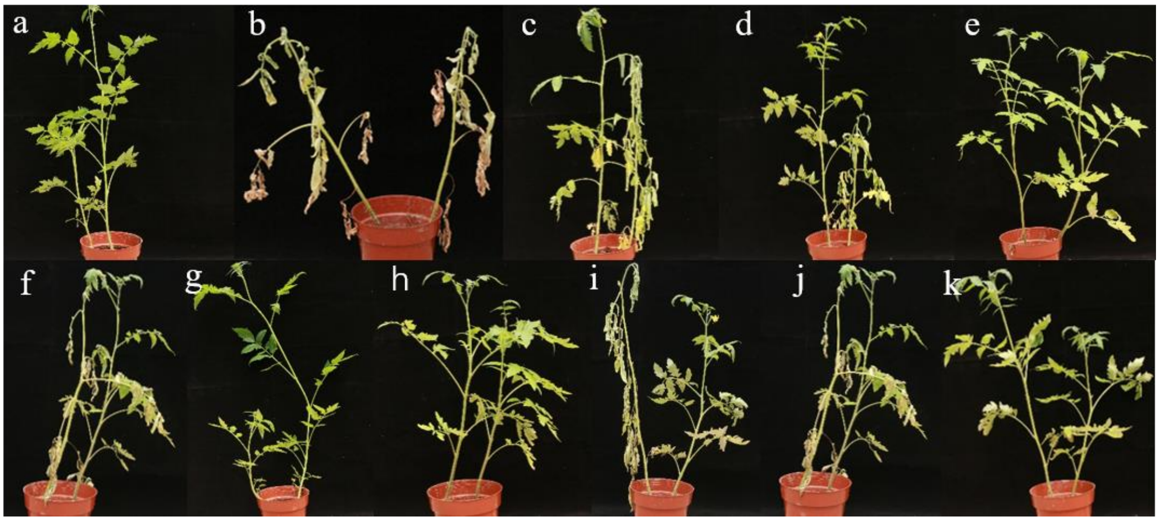

Fig. S2. Effect of antagonistic bacteria on tomato infection by *R. solanacearum*. The plants were treated with medium (a), *R. solanacearum* GMI1000(eGFP) (b), the mixture of GMI1000(eGFP) and biocontrol agent GZ-33 at 1:1 (v/v) (c), 1:2.5 (v/v) (d) and 1:5 (v/v) (e), the mixture of GMI1000(eGFP) and biocontrol agent GZ-34 at 1:1 (v/v) (f), 1:2.5 (v/v) (g), and 1:5 (v/v) (h), the mixture of GMI1000 (eGFP) and biocontrol agent GZ-39 at 1:1 (v/v) (i), 1:2.5 (v/v) (j) and 1:5 (v/v) (k). Each experiment was performed at least three times in triplicate.

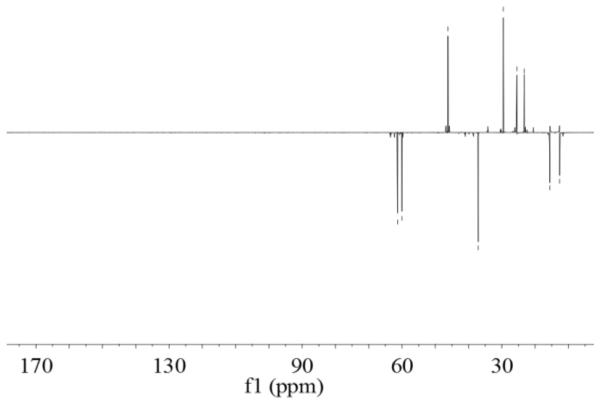

Fig. S3. DEPT135 spectra of cyclo(L-Pro-D-Ile).

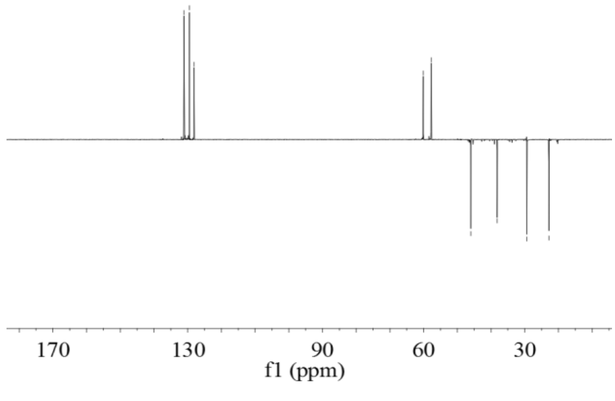

Fig. S4. DEPT135 spectra of cyclo(L-Pro-L-Phe)

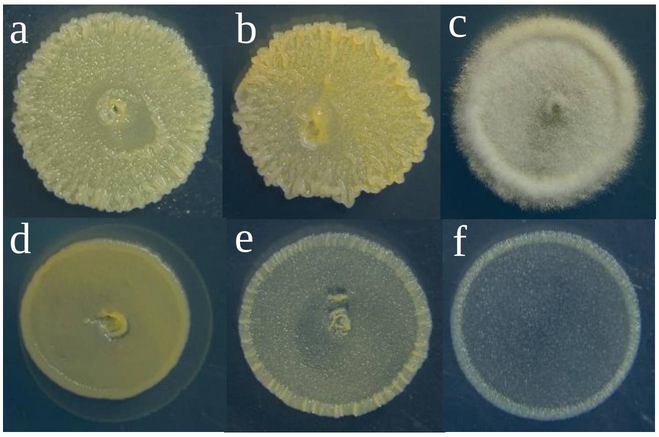

Fig. S5. Effect of the ethyl acetate extract of GZ-34 on the sexual integration of *S. scitamineum*. (a) Ss17 (MAT-1). (b) Ss18 (MAT-2). Ss17 (MAT-1) and Ss18 (MAT-2) were mixed and treated with 0 µl (c), 3 µl (d), 4.5 µl (e) and 6 µl (f) of the ethyl acetate extract of *E. coli* GZ-34, respectively. Each experiment was performed at least three times in triplicate.
